# Factors Affecting Sustainability-Related Career Expectations among Engineering Undergraduates in China: An Empirical Study Based on a Modified College Impact Model

**DOI:** 10.3390/bs14050409

**Published:** 2024-05-14

**Authors:** Wenjing Yuan, Yonghong Ma, Yichu Deng, Xianwei Liu

**Affiliations:** 1 School of Public Administration, Institute of Higher Education, Beihang University, Beijing 100191, China; 09225@buaa.edu.cn (W.Y.); myhong@buaa.edu.cn (Y.M.); 2 Publicity Department, Beijing University of Technology, Beijing 100124, China

**Keywords:** engineering undergraduates, sustainability-related career expectations, curricular emphasis, curricular instruction, sustainability-related career self-efficacy, gender, college impact model

## Abstract

The international engineering education community has reached a consensus regarding the need to enhance engineering students’ awareness of and capability to provide sustainable services in their future careers. Based on a modified college impact model, this study analyzed the impacts of curricular emphasis, curricular instruction, and sustainability-related career self-efficacy on the sustainability-related career expectations of engineering students and investigated the moderating effects of gender on the relationships among the research variables. The results show that both curricular emphasis and curricular instruction have direct positive effects on the sustainability-related career expectations of engineering students; sustainability-related career self-efficacy plays a partial mediating role in this process; and gender significantly moderates the influence of curricular emphasis and curricular instruction on sustainability-related career expectations. The findings of this study provide empirical evidence that can be used by higher education institutions and engineering educators to enhance the belief of engineering students in their ability to solve sustainability-related issues in their future careers and promote the diversification of engineering education.

## 1. Introduction

Sustainability refers to the achievement of a long-term balance among environmental services, economic development, and social welfare, while sustainable development is the approach and means used to achieve sustainability [1]. The pivotal role of engineering and technology in the tasks of addressing and resolving global challenges, such as environmental pollution, disease transmission, resource consumption, climate change, and poverty, has been widely recognized. However, due to the complexity and urgency of global challenges, continuous support and investment in the cultivation of future engineers remain necessary to promote the development of innovative solutions to sustainability issues [2]. Higher engineering education bears significant responsibility for the enhancement of sustainability awareness and the knowledge, skills, and values of future engineers, as well as driving society and industry toward sustainable development [3]. Internationally recognized engineering professional accreditation standards have stipulated that the possession of sustainability literacy is part of the prerequisites for engineering degrees. For example, the Chinese Engineering Education Accreditation Association requires that engineering graduates should possess “an ability to design solutions for complex engineering problems and innovatively design systems, components or processes that meet specific needs with societal, public health, safety, legal, cultural and environmental considerations”, “an ability to apply reasoning informed by contextual knowledge to assess societal, health, safety, legal and cultural issues and consequent responsibilities relevant to professional engineering practice”, and “an ability to understand and evaluate the impact of professional engineering solutions in environmental and societal contexts and demonstrate knowledge of and need for sustainable development” [4].

In fact, sustainability and sustainable development are not new concepts in higher engineering education practices. The cultivation of future engineers who can implement sustainable development strategies and promote the welfare of the majority of people is a major trend in the current reform and development of higher engineering education and teaching [5]. Since the 1990s, some top engineering universities in China have actively promoted the practice of integrating sustainability into engineering education as well as the formation of sustainable universities. For instance, Tsinghua University was the first to propose the “Green University” concept in China, while Tongji University developed the vision of “building a world-class university oriented toward sustainable development” [6]. Despite China having the largest higher engineering education system in the world [7], existing empirical studies on the development of sustainable knowledge, values, and attitudes among engineering students have been conducted mainly in Western countries and regions [8]. On the one hand, the majority of studies have concentrated on summarizing and analyzing the typical programs and approaches implemented by higher education institutions (HEIs) to integrate sustainability into teaching and curriculum [9,10]; on the other hand, evaluating the effectiveness of sustainable education in the engineering field and investigating the impacts of relevant courses and teaching methods on college students’ sustainable learning outcomes have emerged as a major concern in the literature for higher engineering education [11].

Individual careers are external manifestations of an individual’s self-realization and serve as a bridge between individuals and society [12]. A huge number of empirical studies in the fields of education and psychology have been conducted to explore college students’ career expectations [13]. Among them, influential theories such as the college impact model (CIM) were widely utilized to establish a logical connection between individual characteristics, educational experience, and socio-psychological factors with college students’ career outcomes [14,15]. Scholars have reached a consensus regarding the need to enhance engineering students’ awareness of and capability to provide sustainable services in their future careers [16]. However, further studies are still needed to determine if engineering students expect to address sustainability-related issues in their future careers, as well as how education and individual factors influence their sustainability-related career expectations. Moreover, increasing gender diversity and equity in engineering education and producing sufficient numbers of highly qualified female engineers are critical for achieving sustainable development goals [17]. A growing number of studies have contributed to our knowledge about the gender gap in the relationship between engineering students’ learning experiences and outcomes [18,19,20,21]. We need to seek further evidence and consider whether engineering students’ sustainability learning outcomes differ by gender according to what is taught and how. 

Accordingly, the major objective of this study is to employ a modified CIM to (1) examine the impacts of two key components of curricular experiences (i.e., curricular emphasis and curricular instruction) on engineering students’ sustainability-related career expectations; (2) investigate the mediating role of sustainability-related career self-efficacy in the relationship between curricular experiences and sustainability-related career expectations; and (3) test the moderating effects of gender on the relationships among the variables included in the modified college impact model. This study is one of the few attempts to evaluate engineering students’ sustainability learning outcomes based on the perspectives of the CIM. By doing so, this study adds to the literature by introducing a college-impact view of sustainability learning outcomes. Specifically, the findings of this study broaden the CIM’s potential for use in promoting engineering undergraduates’ sustainability attitudes and behaviors and clarify its boundary conditions by highlighting the moderating role of gender as a critical and profound topic in engineering education studies. More importantly, with a better understanding of what drives sustainability-related career expectations, we expect this study to provide additional insight into ways of enhancing the belief of engineering students in their ability to solve sustainability-related issues in their future careers and promote the diversified development of higher engineering education.

## 2. Theoretical Foundation and Research Hypotheses

### 2.1. Modified College Impact Model

The CIM was developed on the basis of Astin’s input–environment–output model [22], which aims to explain the complex connections among students’ backgrounds, learning experiences, and learning outcomes [23]. Since its proposal, CIM has primarily been used to explain students’ choices, persistence, and learning outcomes in specific engineering disciplines [24,25]. CIM claims that an individual’s background factors and characteristics (such as gender, ethnicity, health status, personality traits, and the socioeconomic status of his or her family) may affect his or her learning experiences (e.g., curriculum, instruction, and classroom). Furthermore, such experiences may affect learning outcomes (e.g., career expectations) both directly and indirectly through motivational and psychosocial processes (e.g., self-efficacy). Astin stated that the educational experience “must elicit sufficient student effort” [26] (p. 522), which is mostly dependent on “how motivated the student is” [26] (p. 522). Several recent studies have provided clear evidence regarding the possible motivational and psychosocial processes that underlie engineering undergraduates’ educational experiences and their learning outcomes [27,28].

**Figure 1:**
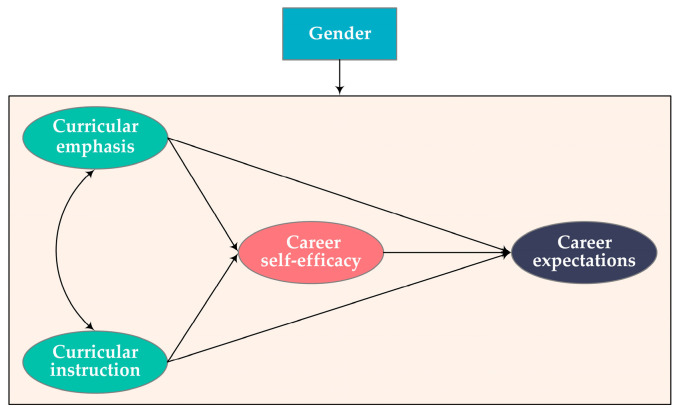
Research framework.

As an important aspect of students’ learning outcomes and a strong predictor of their actual career choices [29,30], career expectations refer to the goals that the individual expects to achieve through his or her career [31]. Accordingly, sustainability-related career expectations are defined here as engineering undergraduates’ desire and aspirations to solve sustainability-related issues through their future occupations. Researchers have found that the key variables included in the CIM have significant impacts on engineering undergraduates’ sustainability-related knowledge, attitudes, and behaviors [28]. Moreover, with a sociocultural perspective on learning, some research based on CIM investigated the differential relationship between college experiences and engineering undergraduates’ learning outcomes based on their gender [18,32]. Following the perspectives of the CIM and its findings, this study explicitly tests the mechanism and boundary conditions of the relationship between curricular experiences and engineering students’ sustainability-related career expectations by incorporating sustainability-related career self-efficacy as a mediator and gender as a moderator in the CIM. Figure 1 shows the research framework for this study. 

### 2.2. Curricular Experiences and Sustainability-Related Career Expectations

According to the CIM, individual student experiences are the dominant factors in shaping student career interests, career preferences, and career choices [23,24]. Curricular experiences formulate engineering undergraduates’ perceptions of their curricular environment as a supportive source that can enable them to solve sustainability-related issues in their future careers, including curricular emphasis and curricular instruction. The former refers to the extent of sustainability-related knowledge and skills emphasized in their engineering courses, and the latter mainly reflects the kind of instructional strategies and activities utilized to deliver sustainability-related knowledge and skills to students throughout their engineering programs [33]. The engineering education community has recognized the fact that incorporating sustainability knowledge and information into the educational system in appropriate ways can enhance engineering students’ capabilities to contemplate new engineering solutions while taking into account sustainable production and consumption [34,35]. Many studies have provided empirical support for the predictive role of curricular experiences in the development of undergraduate students. For example, a longitudinal data-based study in the U.S. revealed that high-impact college experiences significantly influenced college students’ career planning and decision making [14]. Other studies found that college students who participated in service-learning courses had stronger desires to engage in sustainability-related work after graduation [36,37,38]. A recent study based on data drawn from Chinese engineering undergraduates further demonstrated that both curricular emphasis and curricular instruction exert significant influence on undergraduates’ sustainable literacy [28]. Therefore, this study infers that students’ curricular experiences regarding the sustainability content emphasized in courses, as well as instructional activities related to sustainability, can promote their interest in sustainability and their willingness to address sustainability-related issues in their future careers. Accordingly, the following hypotheses are proposed in this study:

**Hypothesis** **1** **(H1).** *Curricular emphasis has a significant positive impact on the sustainability-related career expectations of engineering undergraduates.*

**Hypothesis** **2** **(H2).** *Curricular instruction has a significant positive impact on the sustainability-related career expectations of engineering undergraduates.*

### 2.3. The Mediating Role of Sustainability-Related Career Self-Efficacy

Self-efficacy is the core element of social cognitive theory and the so-called critical motivational factor in the CIM model [22,39]. According to social cognitive theory, it refers to an individual’s belief system with respect to their ability to organize and execute specific action plans effectively in a given situation to achieve a predetermined behavioral goal [40]. Specifically, it focuses on individuals’ judgments, beliefs, or self-assurance and feelings with regard to their capability to perform a specific activity at a certain level, namely, their sense of competence, confidence, and self-esteem in the face of a task [40]. Career self-efficacy is the manifestation of self-efficacy in the field of career development. In this study, sustainability-related career self-efficacy is defined as individuals’ judgments regarding their capability to address sustainability-related issues in their future careers. The educational environment provides students with necessary sources of information to build up their career self-efficacy, such as vicarious experiences, verbal persuasion, emotional arousal, and ways of shaping their sense of control [41].

Several theories have highlighted the close relationship between career self-efficacy and the formation of individual career expectations. For example, the social cognitive theory posits that self-efficacy beliefs promote career expectations because they serve as a functional mechanism for maximizing the motivating potential of career expectations [42]. Furthermore, social cognitive career theory suggests that both self-efficacy beliefs and career expectations serve as incentives for job search implementation [43] and assumes that career self-efficacy bridges positive learning experiences and students’ future career behavior in which they anticipate positive outcomes [44], as individuals who are more confident in their abilities are optimistic about their likelihood of obtaining what they value through their careers [45]. Accordingly, engineering undergraduates’ experiences with their curriculum and instruction activities affect how they view their career path, which, in turn, triggers corresponding attitudes, intentions, and actual behaviors toward sustainability-related careers [46]. In the field of sustainable education, the literature repeatedly indicates that the cultivation of systems thinking, interdisciplinary thinking, and critical and creative thinking through educational activities is helpful in enhancing students’ sustainability-related career self-efficacy, thus motivating students to become responsible and proactive individuals and encouraging them to believe that they have the ability in their future careers to choose, execute, and control their actions in a manner that is conducive to the realization of sustainable goals [47]. Therefore, the following hypotheses are proposed in this study:

**Hypothesis** **3** **(H3).** *Sustainability-related career self-efficacy mediates the relationship between curricular emphasis and sustainability-related career expectations.*

**Hypothesis** **4** **(H4).** *Sustainability-related career self-efficacy mediates the relationship between curricular instruction and sustainability-related career expectations.*

### 2.4. The Moderating Role of Gender

To promote sustainable development and increase the diversity of participation in the engineering field, gender issues have always been a significant focus of engineering education research [48,49]. One advantage of CIM over other student development models is that CIM explicitly incorporates individual characteristics, including gender, into its empirical framework of an individual’s career development and choices. According to sociocultural learning perspectives, gender categories have varying social meanings across cultures, social settings, and time periods [50]. These social meanings are not physical characteristics of individuals but affect how they are viewed and treated (as well as the personal beliefs and biases that they develop) and whether they are included in a particular community [50]. CIM researchers further pointed out that male and female engineering students who took the same curriculum and received the same instruction may obtain varied learning results depending on how they perceive and value the specific topics covered in their curriculums [18,28]. Generally, due to the traditional male-dominated nature of the engineering field and its significant gender imbalance compared to other fields [51], women often lack the necessary career motivation, role model references, and resource support for their educational engagement and career development in engineering [52]. This situation further leads to a lack of confidence, interest, and persistence in pursuing degrees and careers in engineering on the part of women [53,54]. Previous studies have primarily examined gender differences in the students’ learning experiences, self-efficacy, and career expectations included in the modified CIM [18,28,55,56]. For example, Zhao et al. [28] found that learning experiences enhanced engineering students’ sustainable agency, but this was particularly true for males. The reason for this may be that although women are at a disadvantage in the engineering field, the career expectations of female engineering undergraduates tend to be more altruistic and prosocial than those of their male counterparts due to differences in social gender role expectations [57]; furthermore, women are more likely to believe that they have an obligation to address sustainability-related issues in their future careers and are more willing to do so [51]. These findings and perspectives indicate that the gender role of females may suppress the influence of learning experiences and self-efficacy on their sustainability-related career expectations. In addition, due to societal gender role expectations, female engineering students are willing to address sustainability-related issues in their future careers, regardless of their levels of educational experience and self-efficacy. Among male engineering students, however, willingness to promote sustainable development in their future careers is more strongly influenced by relevant educational experiences and self-efficacy [58]. Accordingly, the following hypothesis is proposed in this study:

**Hypothesis** **5** **(H5).** *Gender moderates the relationships between curricular experiences, career self-efficacy, and sustainability-related career expectations, such that these relationships are stronger among male students than female students.*

## 3. Methodology

### 3.1. Sample

The data used in this study were drawn from the “Engineering Education and Sustainable Development Survey”, which was jointly conducted by the Capital Engineering Education Development Research Base of Beijing University of Technology and the Higher Engineering Education Research Center of Beihang University in 2021. The survey targeted senior engineering undergraduates at 14 “Double First-Class” science and engineering universities in Beijing, Shanghai, Tianjin, Jiangsu, Hubei, and Shaanxi. On the survey instruction page, we told participants about the purpose of this study, the voluntary and confidential nature of their participation, and other concerns that they needed to be aware of in order to complete the questionnaire items. A total of 2100 questionnaires were distributed. Of the 1987 questionnaires returned, 1804 were valid. Among the participants, 589 were female, accounting for 32.6% of the total, and 1215 were male, accounting for 67.4% of the total.

### 3.2. Measurement

The questionnaire used in this study consisted of the following two parts: the basic background information of the participants and the scales of the research variables. The four scales and corresponding items included in this study were all drawn from mature scales published in peer-reviewed journals and were verified by empirical studies across contexts and cultures.

The two measures of curricular experiences were adapted from empirical studies based on the CIM [33]. Specifically, the curricular emphasis scale consisted of four items that asked engineering undergraduates to indicate the extent to which sustainability-related knowledge and skills were emphasized in their formal courses on a five-point Likert scale (1 = little emphasis to 5 = very strong). A sample item was “The value of gender, racial/ethnic, or cultural diversity in engineering”. The items on the curricular emphasis scale showed strong reliability (Cronbach’s α = 0.887). The curricular instruction scale consisted of four items that required engineering undergraduates to report the frequency with which different teaching strategies for sustainability were used throughout their engineering programs on a 5-point Likert scale (1 = never to 5 = always). A sample item was “Introduced how sustainability is connected to engineering”. The curricular instruction items demonstrated excellent reliability (Cronbach’s α = 0.930).

In accordance with the theme and context of this study, a four-item engineering undergraduates’ sustainability-related career self-efficacy scale was derived from the abbreviated career self-efficacy scale, which has been widely used in career development research [59]. Engineering undergraduates were required to evaluate their levels of confidence in their abilities to address sustainability-related issues in their future careers on a 5-point Likert scale (1 = strongly disagree to 5 = strongly agree). A sample item was “My learning experiences have equipped me with the ability to address sustainability-related issues in my future career”. The reliability of the scale (Cronbach’s α = 0.760) was confirmed.

A 10-item sustainability-related career expectations scale was designed based on the 10 major challenges facing humanity at present and over the next 50 years, as proposed by Richard E. Smalley, a Nobel laureate in chemistry [60]. These 10 challenges reflected three major dimensions of sustainability-related issues (i.e., economy, society, and environment) with which college students are relatively familiar. Specifically, the 10 items were energy (supply and demand issues), water resources (shortages and pollution issues), food supply, environmental degradation, poverty and resource allocation, climate change, terrorism and war, diseases, development opportunities for future generations, and development opportunities for women and ethnic minorities. Engineering undergraduates were asked to describe their willingness to solve the aforementioned issues in their future careers on a 5-point Likert scale (1 = very unwilling to 5 = very willing). The career expectations scale demonstrated a high reliability (Cronbach’s α = 0.929).

### 3.3. Data Analysis

In this study, the SPSS 26.0 statistical software was used for the basic descriptive statistics, reliability analysis, and correlation analysis; the AMOS 26.0 statistical software was used to test the paths among the research variables, following the two-step strategy for structural equation model (SEM) analysis [61]. The first step involved verifying the fit and validity of the measurement model, while the second involved constructing and testing a structural model for the relationships among the research variables. The indices used to evaluate the model’s goodness of fit included the following: the ratio of chi-square to the degree of freedom (χ^2^/df < 5), the goodness-of-fit index (GFI > 0.90), adjusted goodness-of-fit index (AGFI > 0.90), comparative fit index (CFI > 0.90), incremental fit index (IFI > 0.90), Tucker–Lewis index (TLI > 0.90), standardized root mean square residual (SRMR < 0.08), and the root mean square error of approximation (RMSEA < 0.08) [62]. With the assistance of AMOS 26.0, bootstrapping analysis (5000 bootstrap samples) was performed to estimate the indirect effects of curricular experiences on sustainability-related career expectations through sustainability-related career self-efficacy and the corresponding 95% confidence intervals (CIs). If the 95% CI did not include zero, it indicated a significant direct or indirect effect [63]. Furthermore, multigroup SEM analysis was used to determine the moderating role of gender in the research model. 

## 4. Results

### 4.1. Measurement Model

**Figure 2:**
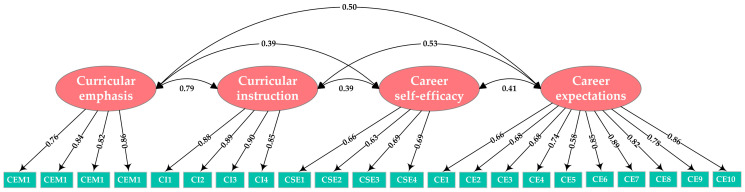
Results of the CFA.

Before conducting the structural model analysis, it was necessary to determine the fit of the factor structure. Specifically, a confirmatory factor analysis (CFA) was initially performed on a measurement model featuring four latent variables and twenty-two observed indicators. The fit indices of the test model were *χ*^2^ = 745.636, *df* = 190, *χ*^2^/*df* = 3.924, GFI = 0.964, AGFI = 0.952, CFI = 0.980, NFI = 0.973, IFI = 0.980, SRMR = 0.038, and RMSEA = 0.040 [90% CI: 0.037, 0.043]. These results indicated that the measurement model exhibited a relatively good fit with the data [62]. As demonstrated in Figure 2, all items exhibited significant standard loadings on their respective constructs and exceeded the threshold of 0.5 [64].

As the data were collected using the self-reported survey in this study, Harman’s one-factor test was used to check for the problem with common method variance (CMV) [65]. The results of the confirmatory factor analysis showed that the fit indices for the one-factor model were far from reaching the standard thresholds (*χ*^2^ = 7103.342, *df* = 196, *χ*^2^/*df* = 36.242, GFI = 0.663, AGFI = 0.566, CFI = 0.747, NFI = 0.742, IFI = 0.748, SRMR = 0.134, RMSEA = 0.140 [90% CI: 0.137, 0.143]), thus indicating that the one-factor model exhibited a very poor fit with the data. Furthermore, the *χ*^2^ difference test revealed that the fit of the measurement model with the data was significantly superior to that of the one-factor model (Δ*χ*^2^ = 6357.706, Δ*df* = 6, *p* < 0.001). Therefore, it can be inferred that CMV had little impact on this study.

Following the CFA, the composite reliability (CR), average variance extracted (AVE), and correlation coefficients of each construct were calculated. As shown in Table 1 the CR values of each variable were all above the standard of 0.70 [66], and the AVE values also satisfied the criterion of 0.50 [67]. Additionally, significant correlations were observed among the variables, and the square root of the AVE was greater than the correlation coefficients between each pair of variables [67]. These results indicate that the measures in this study exhibited excellent convergent and discriminant validity.

### 4.2. Structural Model

**Figure 3:**
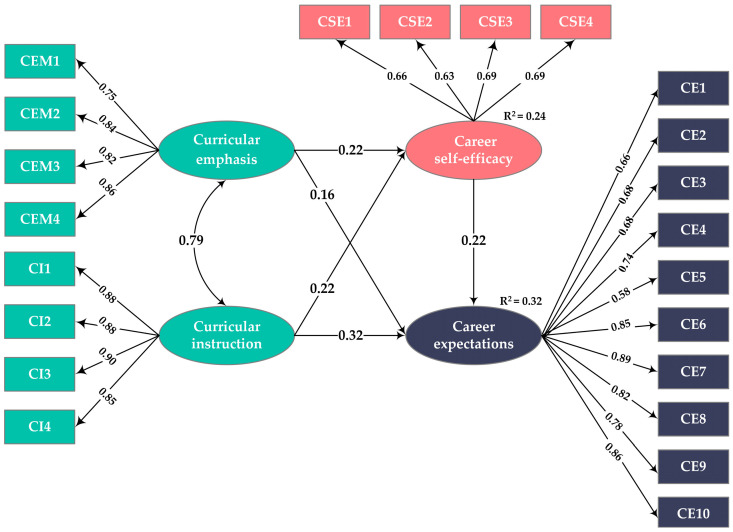
Results of the SEM analysis.

After the analysis of the measurement model, a structural model for the relationships among the research variables was constructed. The fit indices of the model were *χ*^2^ = 745.636, df = 190, *χ*^2^/*df* = 3.924, GFI = 0.964, AGFI = 0.952, CFI = 0.980, NFI = 0.973, IFI = 0.980, SRMR = 0.038, and RMSEA = 0.040 [90% CI: 0.037, 0.043], thus indicating that the proposed structural model exhibited an excellent fit with the data. The model and its test results are detailed in Figure 3.

According to Figure 3, curricular emphasis had a significant positive direct effect on both sustainability-related career self-efficacy (*β* = 0.223, *t* = 4.460, *p* < 0.001) and sustainability-related career expectations (*β* = 0.158, *t* = 3.859, *p* < 0.001); similarly, curricular instruction also had a significant positive direct effect on sustainability-related career self-efficacy (*β* = 0.216, *t* = 4.396, *p* < 0.001) and sustainability-related career expectations (*β* = 0.324, *t* = 7.921, *p* < 0.001). Additionally, the positive direct effect of sustainability-related career self-efficacy on sustainability-related career expectations was significant (*β* = 0.218, *t* = 8.010, *p* < 0.001). Therefore, H1 and H2 were supported.

Using the bias-corrected percentile bootstrap method, the 95% CIs of the indirect effects were estimated by extracting 5000 bootstrap samples to determine the mediating effects of sustainability-related career self-efficacy in the relationship between curricular experiences and sustainability-related career expectations. As shown in Table 2, both curricular emphasis and curricular instruction had significant direct and indirect effects on sustainability-related career expectations (all 95% CIs did not contain 0), supporting the fact that sustainability-related career self-efficacy played a partial mediating role in the relationship between curricular experiences and career expectations. Based on these results, H3 and H4 were verified.

### 4.3. Moderating Effects

Multigroup SEM analysis was used to test the moderating effects of gender on the structural relationships in the proposed model. Specifically, the first step was to examine the difference between an unconstrained model (that allowed for free estimation of the paths among the variables) and a constrained model (that required all paths among the variables to be equal). In both models, factor loadings were held constant to ensure that the testing structure was the same between the male and female groups; however, the error terms were allowed to be estimated freely in both models. If a significant difference in the *χ*^2^ values was observed between the two models, it indicated that gender had a moderating effect on one or more paths [66]. The *χ*^2^ difference test revealed that the constrained model (*χ*^2^ = 1130.512, *df* = 403) and unconstrained model (*χ*^2^ = 1118.981, *df* = 398) were statistically different (Δ*χ*^2^ = 11.531, Δ*df* = 5, *p* < 0.05), providing evidence for the moderating role of gender on the structural paths of the proposed model. 

To further detect the moderating effects of gender on specific paths, a series of *χ*^2^ difference tests were performed on the constrained model and five unconstrained models (each was allowed only the path being tested to be estimated freely) [68]. As detailed in Table 3, gender had a significant moderating effect on two out of the five paths in the model. Specifically, the impact of curricular emphasis on sustainability-related career expectations was weaker for female undergraduates (*β* = 0.097, *t* = 1.902, *p* > 0.05) than for male undergraduates (*β* = 0.204, *t* = 4.665, *p* < 0.001). Similarly, the effect of curricular instruction on sustainability-related career expectations was lower for female undergraduates (*β* = 0.229, *t* = 4.389, *p* < 0.001) than for male undergraduates (*β* = 0.359, *t* = 8.554, *p* < 0.001). Therefore, H5 was partially supported.

## 5. Discussion and Implications

With a modified CIM framework, this study investigated the structural relationships between two facets of curricular experiences (curricular emphasis and curricular instruction), sustainability-related career self-efficacy, and sustainability-related career expectations, as well as the moderating role of gender on these relationships among a sample of senior engineering undergraduates in China. The primary findings of this study are presented and discussed below.

In line with the perspectives of CIM, this study revealed that both curricular emphasis and curricular instruction exert significant and positive effects on the sustainability-related career expectations of engineering undergraduates. These results are aligned with previous studies supporting the idea that the incorporation of sustainability-related content and diversified instructional strategies into formal education contributes to enhancing engineering students’ responsibility and willingness to act in a sustainable manner in their future lives and occupations [28,69]. As mentioned, sustainability-related educational experiences provide crucial knowledge, skills, and information for enhancing students’ ability to solve sustainability issues through direct or indirect experiences that occur in the higher education context, which play a fundamental role in shaping their occupational values, according to the CIM [70].

As expected, this study demonstrated that both curricular emphasis and curricular instruction also indirectly influenced sustainability-related career expectations through the partial mediation of sustainability-related career self-efficacy. This finding indicated, given that students are active agents, that their self-efficacy serves as an important bridge between external sustainable education and individuals’ beliefs in sustainability and literacy development [71]. Specifically, sustainability-related career self-efficacy can further help students strengthen their confidence and sense of mission with regard to solving sustainability issues, thus increasing their willingness to perform challenging learning and work tasks in their socialization process; and helping them actively link the professional knowledge and skills they have acquired with real or potential sustainability-related problems; and encourage them to actively seek resources, paths, and strategies to solve these issues. Students can, thus, maintain positive and optimistic attitudes toward their ability to solve sustainability-related issues, set higher goals for their future careers, and link these positive beliefs regarding their ability to solve sustainability-related issues with their future career development [72,73].

This study further revealed the moderating role of gender on the structural model. Specifically, multigroup SEM analysis revealed that both the impacts of curricular emphasis and curricular instruction on sustainability-related career expectations were significantly stronger among male engineering undergraduates than their female counterparts. These findings imply that current sustainability educational and teaching activities have substantial impacts on the development of sustainability-related career expectations among male engineering undergraduates, while among female students, the effects of existing activities seem to be limited [74]. On the one hand, males can access and obtain necessary learning resources more easily than females in the male-dominated engineering field. On the other hand, “women who decide to enter the field of STEM show a very strong expectation that they can make the world a better place” due to the traditional gender roles defined in society [58] (p. 168). 

Our findings provide the following theoretical and pragmatic contributions. Theoretically, this study contributes to the literature on engineering undergraduates’ sustainability-related career expectations using a modified CIM framework. It fills a void in prior research that neglected the significance of curricular experiences in explaining engineering undergraduates’ sustainability-related learning outcomes [75,76]. Additionally, by incorporating gender as a moderator into the CIM, this study provides a more nuanced understanding of the mechanisms and conditions of the relationships between curricular experiences, self-efficacy, and sustainability-related learning outcomes and offers new evidence for gender issues within the male-dominated engineering education context [18]. 

This study also outlines practical insights for policymakers, HEIs, and engineering educators to enhance sustainability-related education activities, thus preparing engineering graduates to successfully address sustainability-related issues in their careers. First, a lack of qualified teachers is still one of the most significant barriers to engineering undergraduates’ sustainability-related learning across countries [28,77,78]. Therefore, HEIs and engineering programs should strengthen the training of teachers, enable them to master sustainability-related knowledge and theoretical systems, understand the intrinsic connection between sustainability and engineering, and enhance the skills and techniques involved in disseminating sustainability knowledge. Second, the cultivation of engineering undergraduates’ positive psychological qualities with regard to sustainability-related careers should be incorporated into the curriculum system, thus helping students genuinely perceive the severity and urgency of sustainability-related issues and the close relationships between sustainability, themselves, and engineering. Third, the traditionally male-dominated engineering fields often overlook women’s experiences and emotions [29]. Therefore, it is vital to develop more inclusive and interactive curricular activities that focus on “females and engineering” and “females and sustainability” to promote the establishment of supportive networks for female students.

## 6. Conclusions and Directions for Future Studies

All in all, based on a modified CIM, this study provides empirical evidence for the structural relationships among sustainability-related curricular experiences, self-efficacy, and engineering undergraduates’ sustainability-related career expectations, as well as the moderating role of gender in these relationships. Therefore, the CIM might serve as a critical meta-framework for exploring engineering undergraduates’ sustainability-related learning outcomes. However, there are several limitations that should be taken into consideration in future research. Similar to other empirical studies, this one relied on self-report surveys and cross-sectional data, which might have led to a response bias and endogeneity problem. Thus, future studies should avoid these problems by adopting a longitudinal design and a cross-lagged approach. Additionally, we recommend that other critical mediators and moderators be further explored based on the CIM in future research due to the global diversity of engineering education.

## Figures and Tables

**Table 1:** Reliability, validity, and the correlation matrix.

Variables	M	SD	CR	AVE	1	2	3	4
1. Curricular emphasis	4.077	0.652	0.889	0.668	*0.817*			
2. Curricular instruction	3.929	0.741	0.930	0.770	0.725 ***	*0.877*		
3. Career self-efficacy	4.059	0.618	0.762	0.445	0.330 ***	0.335 ***	*0.667*	
4. Career expectations	3.959	0.677	0.930	0.576	0.475 ***	0.514 ***	0.349 ***	*0.759*

Diagonal elements (in italics) are the square root of the AVEs; *** *p* < 0.001.

**Table 2:** Results of bootstrapping.

Paths	Bootstrapping		95% Bias-Corrected CI	
	Effect	Boot S. E.	Boot LLCI	Boot ULCI
CEM → CSE	0.223 ***	0.065	0.098	0.352
CEM → CE	0.158 ***	0.054	0.046	0.260
CI → CSE	0.216 ***	0.061	0.094	0.336
CI → CE	0.324 ***	0.051	0.226	0.426
CSE → CE	0.218 ***	0.033	0.152	0.282
CEM → CSE → CE	0.049 ***	0.015	0.023	0.083
CI → CSE → CE	0.047 ***	0.016	0.020	0.084

CEM = curricular emphasis; CI = curricular instruction; CSE = sustainability-related career self-efficacy; CE = sustainability-related career expectations; LLCI = lower-level confidence interval; ULCI = upper-level confidence interval. *** *p* < 0.001.

**Table 3:** Results of the multigroup SEM analysis.

	Standardized Coefficients		*χ*^2^ (*df*)	Δ*χ*^2^ (Δ*df*)
	Female	Male		
**Constrained Model**			1130.512 (403)	
CEM → CSE	0.174 **	0.240 ***	1127.974 (402)	2.538
CEM → CE	0.097	0.204 ***	1123.626 (402)	6.886 **
CI → CSE	0.191 **	0.242 ***	1129.617 (402)	0.895
CI → CE	0.229 ***	0.359 ***	1122.132 (402)	8.380 **
CSE → CE	0.147 ***	0.246 ***	1126.911 (402)	3.601

CEM = curricular emphasis; CI = curricular instruction; CSE = sustainability-related career self-efficacy; CE = sustainability-related career expectations. ** *p* < 0.01. *** *p* < 0.001.

## Data Availability

Data will be made available upon request.
